# Mathematical Modeling of LDH Nanoparticle Drying: Evaluating Effective Diffusivity and the Role of the Mass Biot Number

**DOI:** 10.1021/acsomega.5c10016

**Published:** 2025-12-20

**Authors:** Luiz D. Silva Neto, Rodolfo Junqueira Brandão, Thais Logetto Caetité Gomes, Lucas Meili, José Teixeira Freire

**Affiliations:** † Drying Center of Pastes, Suspensions, and Seeds, Department of Chemical Engineering, 67828 Federal University of São Carlos (UFSCar), São Carlos, São Paulo 13565-905, Brazil; ‡ Flowlab (Fluid Dynamics Laboratory), Center of Technology, 28112 Federal University of Alagoas, Av. Lourival Melo Mota, s/n, Campus A.C. Simões, Tabuleiro do Martins, Maceió, AL 57072-970, Brazil; § Laboratory of Processes, Center of Technology, Federal University of Alagoas, Av. Lourival Melo Mota, s/n, Campus A.C. Simões, Tabuleiro do Martins, Maceió, AL 57072-970, Brazil

## Abstract

Layered Double Hydroxides (LDH) are an important class of inorganic nanomaterials characterized by variable composition, high porosity, significant surface area, and notable ion-exchange capacity. Although coprecipitation is the most widely used synthesis method, the subsequent drying step is often critically overlooked, as it directly affects particle agglomeration and degradation of the porous colloidal structure. This study evaluated the influence of the drying process on MgAl–CO_3_/LDH synthesis by determining the effective diffusivity (*D*_eff_) and activation energy (*E*_a_). LDHs were synthesized via coprecipitation (Mg/Al ratio = 2:1). Drying kinetics were investigated at 75, 90, and 105 °C, both without and with forced-air convection (1.0 ± 0.1 m/s). Diffusivity coefficients were determined using Fick’s second law. A two-parameter diffusion model, including the mass Biot number (*Bi*_m_), provided a superior fit to the experimental data compared with the one-parameter model, corroborating the strong influence of external drying conditions. *D*_eff_ values ranged from a maximum of 4.00 × 10^–7^ m^2^/s (at 105 °C with convection, *Bi*_m_ = 0.01) to a minimum of 3.40 × 10^–10^ m^2^/s (at 75 °C without convection). The average activation energy ranged from 12.55 to 26.80 kJ/mol, suggesting that the superficial diffusion of liquid molecules along capillary surfaces is the rate-limiting mass-transfer mechanism. These results establish a quantitative basis for optimizing LDH drying parameters. They can serve as a starting point for other drying systems, such as fluidized-bed, rotary, spray, and freeze-dryers.

## Graphical abstract

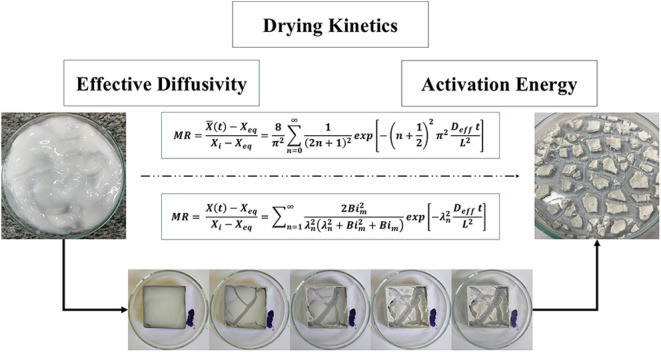

## Introduction

Layered double hydroxides are an important family of nanomaterials distinguished by their diverse chemical compositions and topologies.^1–3^ They are made up of two positively charged lamellae that are held together structurally by interlamellar anions. Exhibiting a crystalline arrangement with variations in the cation ratios and the nature of both cations and anions, LDHs are generally described by the formula [*M*_1–*x*_^II^.*M*_*x*_^III^(OH)_2_]^*x*^·[*A*_*x*/*n*_^*n*–^·*z*H_2_O]^*x*−^, where *M* ^II^ denotes a divalent metal cation, *M* ^III^ a trivalent metal cation, *A* ^*n*–^ an intercalated anion of valence *n*, *z*, the number of water molecules, and *x* the molar ratio *M* ^III^/(*M* ^III^ + *M* ^II^).^4^ These materials exhibit high porosity, significant surface area, and notable ion-exchange capacity.^1^

The first synthesis of layered double hydroxides was reported by Feitknecht in 1933, via the controlled precipitation of aqueous metal cation solutions using a base.^5^ Since then, synthetic methodologies for LDHs have advanced substantially, enabling precise control over their composition, structure, and physicochemical properties. These developments have rendered LDHs highly versatile materials, suitable for a broad spectrum of applications. Over the years, synthesis routes have been extensively investigated and optimized, leading to materials with enhanced performance, improved structural stability, and superior surface characteristics.^3,6,7^

Among the applications, numerous studies have highlighted the potential of tailored LDHs for CO_2_ separation and capture,^8,9^ soil treatment,^10,11^ and catalysis.^12–14^ As a result of these advances, a wide variety of LDH compositions have been synthesized across industrial, pilot, and laboratory scales. To further enhance material properties and scalability, several synthesis methods have been newly developed or adapted from traditional techniques. These continuous improvements reflect the growing scientific interest in optimizing the physicochemical properties of LDHs to meet the demands of increasingly complex applications, thereby consolidating them as materials of strategic importance across various technological fields.^15–18^

The characteristics of nanomaterials—such as specific surface area, pore structure, and particle size and morphology—are strongly influenced by the drying process. The drying phase in the synthesis of LDHs has gotten very little attention despite these developments. The drying process is an operation that involves simultaneous heat and mass transfer processes to remove a solvent, usually water, from a solid, semisolid, or liquid feedstock. Drying nanomaterials is considerably more challenging than drying conventional materials, as the solvent must be carefully removed without compromising the porous microstructure.^19–21^ This stage is critical to the dynamics of solvent evaporation, particle agglomeration, and the collapse of the colloidal porous network.^4,9,22^

To date, no data on the effective diffusivity of the drying process of layered double hydroxides have been found in the consulted literature. Several researchers have described the advantages of studying drying kinetics, including developing a better understanding of controlling drying parameters, understanding transport phenomena associated with processing, and using these insights to control or optimize process variables. In this context, the purpose of this work was to study the phenomena governing the drying process of MgAl–CO_3_ layered double hydroxides. The effective moisture diffusivity and activation energy were determined for each test. Finally, the activation energies were found for each sample thickness. These results can be used to control or optimize the variables of the LDHs drying process, representing a new step toward large-scale applications of this material.

## Material and Methods

### Synthesis of Layered Double Hydroxide

The synthesis of LDHs was performed following the coprecipitation method described by Reichle.^23^ A solution of MgCl_2_·6H_2_O and AlCl_3_·6H_2_O was prepared in deionized water. A second solution, consisting of 50% NaOH and anhydrous Na_2_CO_3_ dissolved in deionized water, was gradually added to the first solution. The reaction was conducted on a mechanical shaker at ambient temperature. The resulting suspension was maintained under constant stirring and temperature for 18 h. Subsequently, the suspension was centrifuged, and the solid materials were washed with deionized water at ambient temperature until the pH reached 10. Finally, the resulting colloidal dispersion was subjected to a drying step. The materials produced were characterized by scanning electron microscopy (SEM) and X-ray diffraction (XRD) (Supporting Information).

### Drying Experiments

Drying experiments were conducted in a forced convection oven at three different temperatures: 75 °C, 90 °C, and 105 °C. The experiments were performed both without and with hot air flow (1.0  ±  0.1 m/s). A 10 g sample of LDH was evenly spread in a Petri dish (approximately 0.002 m in thickness). The mass loss of the samples was periodically monitored using an external analytical balance. At the end of each experiment, the samples were placed in a natural convection oven at 105 °C for 24 h to determine their equilibrium moisture content.

Drying kinetic data were obtained by measuring the sample mass as a function of time at constant temperatures. The dimensionless moisture ratio (MR) was calculated using eq 1, where *X* is the moisture at time *t*, *X*_0_ is the initial moisture content, and *X*_e_ is the equilibrium moisture content. The drying rate was expressed as the change in moisture over time (eq 2), where *X*_*t*+d*t*_ and *X*_*t*_ represent the moisture content at *t* + d*t* and *t*, respectively, with *t* being the drying time.

1 $$\mathrm{MR}=\frac{X\left(t\right)-X_{e}}{X_{0}-X_{e}}$$

2 $$N=\frac{X_{t+dt}-X_{t}}{dt}$$

### Effective Diffusivity and Activation Energy

The diffusivity coefficients were determined using the one-dimensional form of Fick’s second law of diffusion (eq 3). The analytical solution to this partial differential eq (eq 4) was derived by Crank,^24^ assuming a uniform initial moisture distribution, negligible external mass transfer resistance, constant sample thickness (i.e., no shrinkage), and a final equilibrium moisture content close to zero.^25^

3 $$\frac{\partial X\left(z,t\right)}{\partial t}=\frac{\partial }{\partial z}\left[D_{\mathrm{eff}}\frac{\partial X\left(z,t\right)}{\partial t}\right]$$

4 $$\mathrm{MR}=\frac{\mathrm{X̅}\left(t\right)-X_{\mathrm{eq}}}{X_{i}-X_{\mathrm{eq}}}=\frac{8}{\pi^{2}}\sum_{n=0}^{\infty }\frac{1}{{\left(2n+1\right)}^{2}}\exp\left[-{\left(n+\frac{1}{2}\right)}^{2}\pi^{2}\frac{D_{\mathrm{eff}}t}{L^{2}}\right]$$

where *X*_eq_ is the dynamic equilibrium moisture, *X*_*i*_ is the moisture at the initial time (*t* = 0), *n* is the number of terms in the equation, and *L* is the material thickness.

If external resistance to mass transfer is assumed at the particle surface, the boundary condition at *t* > 0 and *z* = *Z* is applied. Based on these, Crank^26^ obtained an analytical solution that resulted in eq 5. The solution of the equation requires the estimation of two parameters, namely the mass Biot number (*Bi*_m_) and the effective diffusivity (*D*_eff_). The eigenvalues (λ_*n*_) are obtained from the transcendental eq (eq 6).^27,28^

5 $$\mathrm{MR}=\frac{X\left(t\right)-X_{\mathrm{eq}}}{X_{i}-X_{\mathrm{eq}}}=\sum_{n=1}^{\infty }\frac{2{\mathrm{Bi}}_{m}^{2}}{\lambda_{n}^{2}\left(\lambda_{n}^{2}+{\mathrm{Bi}}_{m}^{2}+{\mathrm{Bi}}_{m}\right)}\exp\left[-\lambda_{n}^{2}\frac{D_{\mathrm{eff}}t}{L^{2}}\right]$$

6 $${\mathrm{Bi}}_{m}=\lambda_{n}\tan\left(\lambda_{n}\right)$$

The activation energy was determined from the dependence of the effective moisture diffusivity on temperature using an Arrhenius-type eq (eq 7).^27^

7 $$D_{\mathrm{eff}}=D_{0}\exp\left(-\frac{E_{a}}{\mathrm{RT}}\right)$$

where *E*_a_ is the activation energy (kJ/mol), *D*_0_ is a pre-exponential factor equivalent to high-temperature diffusivity (limit diffusion coefficient) (m^2^ /s), *R* is the universal gas constant (kJ/mol·K), and *T* is the absolute temperature (K).

The evaluation of the model fit to the experimental data was performed using the Coefficient of Determination (*R* ^2^, eq 8), the Root Mean Square Error (RMSE, eq 9), and the chi-square statistic (*x* ^2^, eq 10).

8 $$R^{2}=1-\frac{\sum_{i=1}^{N}{\left({\mathrm{MR}}_{\exp,i}-{\mathrm{MR}}_{\mathrm{pre},i}\right)}^{2}}{\sum_{i=1}^{N}{\left(\bar{{\mathrm{MR}}_{\exp,i}}-{\mathrm{MR}}_{\mathrm{pre},i}\right)}^{2}}$$

9 $$\mathrm{REQM}=\sqrt{\frac{1}{N}\sum_{i=1}^{N}{\left({\mathrm{MR}}_{\exp,i}-{\mathrm{MR}}_{\mathrm{pre},i}\right)}^{2}}$$

10 $$x^{2}=\frac{\sum_{i=1}^{N}{\left({\mathrm{MR}}_{\exp,i}-{\mathrm{MR}}_{\mathrm{pre},i}\right)}^{2}}{N-z}$$

where MR_exp_ is the value of the experimental dimensionless moisture, MR_pre_ is the dimensionless moisture value predicted by the model, $\bar{{\mathrm{MR}}_{\exp}}$ is the average value of the experimental dimensionless moisture, *N* is the number of experiments, and *z* is the number of constants in the mathematical model.

## Results and Discussion

### Drying Kinetics

The drying behavior of LDH/MgAl-CO_3_ at different temperatures is presented in Figure 1. Figure 1(a),(b) show the evolution of the dimensionless moisture ratio (MR) over drying time under nonconvective (*v* = 0 m/s) and convective (*v* = 1 m/s) conditions, respectively. The XRD patterns and SEM images obtained are presented in the Supporting Information, in Figures S1 and S2, respectively. In both cases, a continuous decrease in MR is observed, indicating effective moisture removal.

**Figure 1:**
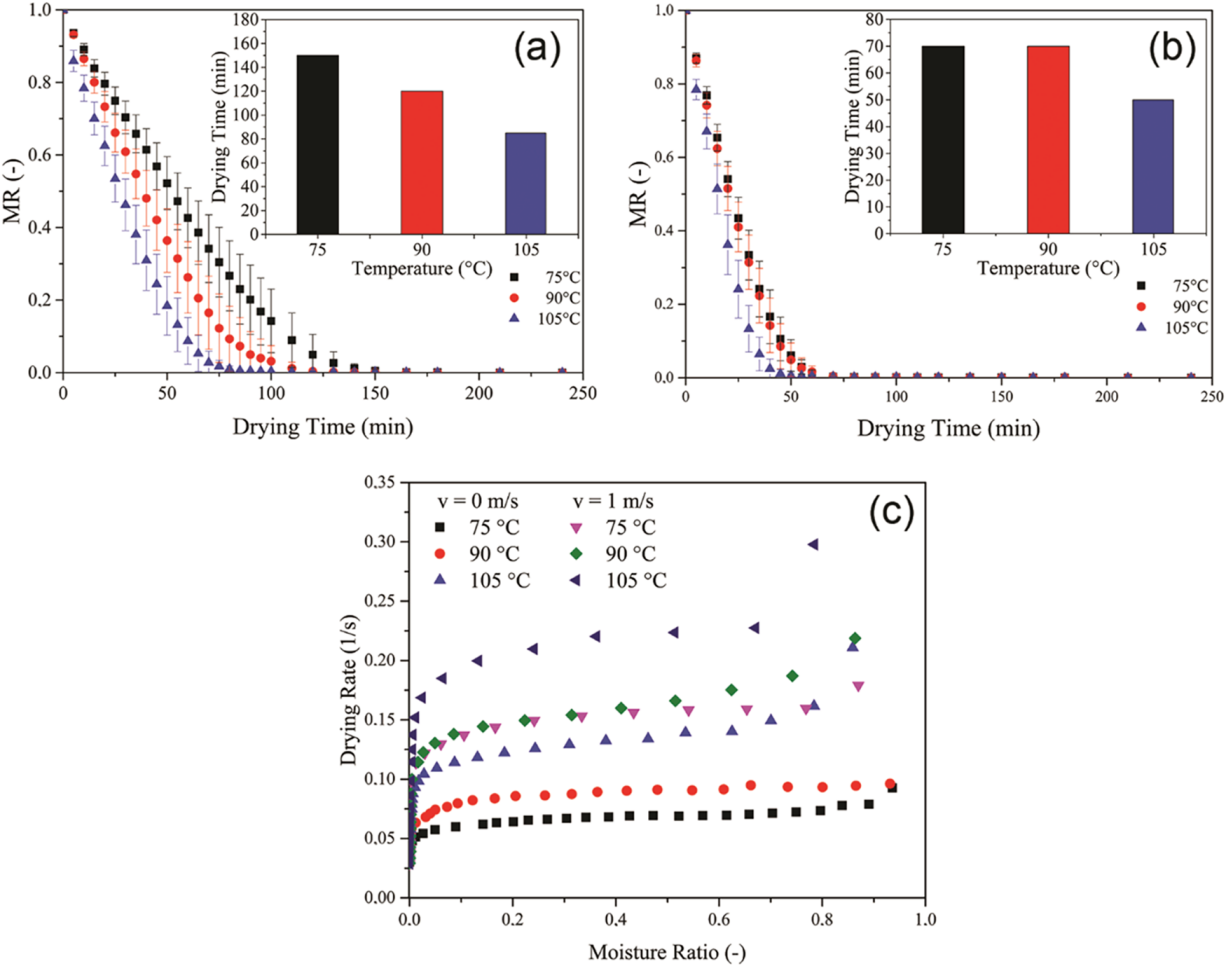
Dimensionless moisture content of LDH/MgAl-CO_3_ as a function of drying time at different temperatures (75, 90, and 105 °C), under (a) absence of convection (*v* = 0 m/s) and (b) forced convection (*v* = 1 m/s). (c) Drying rate as a function of dimensionless moisture ratio.

As expected, higher temperatures resulted in faster drying due to the increased thermal gradient between the material and the surrounding air. This effect is particularly evident under nonconvective conditions, where heat transfer is limited to conduction and natural convection. Figure 1(a) demonstrates that at 105 °C, drying times are significantly shorter compared to 75 °C and 90 °C.

In Figure 1(b), forced convection (*v* = 1 m/s) substantially enhances the drying process. The MR curves display a steeper decline, especially at elevated temperatures. This improvement is attributed to enhanced heat and mass transfer coefficients caused by increased interstitial air velocity. The inset bar charts in both figures confirm that drying time decreases with rising temperature, with more pronounced reductions under convective conditions.

At the beginning of the drying process, a rapid decline in the evaporation rate is observed, primarily due to the high initial moisture content. In this initial phase, heat is mainly used to remove free water, and the drying rate is largely independent of time and suspension thickness.^19,21,29^

The structural characteristics of LDH contribute to this behavior. Due to its relatively high specific surface area, the material exhibits an efficient heat and mass transfer even in small volumes.^29–32^ The irregular flow of fluid through the porous structure promotes elevated interstitial velocity, favoring turbulent transport mechanisms. Moreover, capillary pressure and rapid initial evaporation can induce micropore collapse and structural deformation, leading to the formation of large agglomerates.^29^

### Effective Diffusivity

The effective moisture diffusion coefficient reflects the dehydration capacity of materials under specific drying conditions and is considered one of the most critical parameters for a drying process design.^33^ Table 1 presents the estimated values of effective diffusivity obtained through the diffusive model (eq 7) for each experimental condition. The maximum and minimum values found for the diffusivity were 1.28 × 10^–09^ and 3.40 × 10^–10^ m^2^/s for temperatures of 105 °C with convection and 75 °C without convection, respectively. In convective drying, the increase of *D*_eff_ temperature can result from the activation of water molecules, accelerating their transfer to the material surface. Based on the results obtained, it is observed that, despite the values for the coefficient of determination being greater than 0.9, the adjustment provided by the Diffusive Model with *D*_eff_ constant is not satisfactory. In the Diffusive Model, an analytical solution is used that considers only the internal diffusivity to estimate *D*_eff_ by fitting eq 7 to the experimental kinetic data. Thus, possibly the deviations found between the observed and predicted data may be related to the considerations made to solve the model. Among some of the considerations, there is negligible shrinkage and constant effective diffusivity.^34,35^ It is important to note that the effective diffusion coefficient should not be interpreted only in terms of the molecular diffusion coefficient but rather as a parameter of much more complex definitions.^36^

**Table 1:** Estimated Values for Effective Diffusivity for the Diffusive Model

	with convection			without convection		
temperature (°C)	*D*_eff_ (m^2^/s)	*R* ^2^	*x* ^2^	*D*_eff_ (m^2^/s)	*R* ^2^	*x* ^2^
75	3.40 × 10^–10^	0.9020	0.0106	8.73 × 10^–10^	0.9341	0.0065
90	4.89 × 10^–10^	0.9125	0.0095	9.18 × 10^–10^	0.9405	0.0057
105	7.10 × 10^–10^	0.9346	0.0061	1.28 × 10^–09^	0.9485	0.0042

To investigate the adjustment provided by the diffusion model when the external resistance is considered, a diffusion model with two mass parameters, the dimensionless Biot number (*Bi*_m_) and the effective diffusivity of the liquid (*D*_eff_), was applied (eq 8).^24,27,28^ Table 2 presents the estimated effective diffusivities for each experimental condition.

**Table 2:** Estimated Values for Effective Diffusivity According to the Respective *Bi*_m_

		with convection			without convection		
*Bi*_m_	temperature (°C)	*D*_eff_ (m^2^/s)	*R* ^2^	*x* ^2^	*D*_eff_ (m^2^/s)	*R* ^2^	*x* ^2^
0.01	75	1.16 × 10^–07^	0.9709	0.0031	2.82 × 10^–07^	0.9772	0.0023
	90	1.62 × 10^–07^	0.9733	0.0029	2.94 × 10^–07^	0.9802	0.0019
	105	2.24 × 10^–07^	0.9760	0.0022	4.00 × 10^–07^	0.9792	0.0017
0.1	75	1.19 × 10^–08^	0.9708	0.0031	2.90 × 10^–08^	0.9772	0.0023
	90	1.67 × 10^–08^	0.9733	0.0029	3.03 × 10^–08^	0.9802	0.0019
	105	2.31 × 10^–08^	0.9760	0.0022	4.11 × 10^–08^	0.9792	0.0017
1	75	1.54 × 10^–09^	0.9696	0.0033	3.76 × 10^–09^	0.9760	0.0024
	90	2.17 × 10^–09^	0.9720	0.0030	3.92 × 10^–09^	0.9790	0.0020
	105	3.00 × 10^–09^	0.9751	0.0023	5.33 × 10^–09^	0.9781	0.0018
100	75	3.55 × 10^–10^	0.9070	0.0100	9.07 × 10^–10^	0.9361	0.0063
	90	5.09 × 10^–10^	0.9162	0.0091	9.53 × 10^–10^	0.9426	0.0055
	105	7.36 × 10^–10^	0.9374	0.0059	1.33 × 10^–09^	0.9499	0.0041
Inf	75	3.40 × 10^–10^	0.8982	0.0110	8.73 × 10^–10^	0.9307	0.0069
	90	4.89 × 10^–10^	0.9090	0.0099	9.17 × 10^–10^	0.9374	0.0060
	105	7.09 × 10^–10^	0.9319	0.0064	1.28 × 10^–09^	0.9457	0.0044

Using the diffusive model with two mass parameters, the diffusivity values were higher, going from a magnitude of 10^–10^ to 10^–07^. Comparing Tables 1 and 2, it is observed that this model presents higher values for *R* ^2^ and lower values of RMSE and *x* ^2^, compared to the diffuse model with only one parameter, corroborating the influence of external drying conditions.

Lower values of the *Bi*_m_, indicating that external mass transfer resistance predominates, provided better model fitting. These findings, along with the kinetic and drying rate data, confirm that external drying conditions significantly influence the drying process. For *Bi*_m_ > 100, where internal resistance dominates, the estimated *D*_eff_ values closely approach those obtained using the one-parameter diffusion model. Figure 2 presents the experimental drying kinetics curves for LDHs dried at 75, 90, and 105 °C, along with the predictions from the one- and two-parameter diffusion models, with a Biot number of 0.01. Similar to the diffusion model, the two-parameter model underestimates the dimensionless moisture at the beginning of the process and overestimates it at the end. However, the model with *Bi*_m_ = 0.01 shows a smaller difference between the experimental and predicted data, presenting a better fit. Furthermore, *D*_eff_ is an effective parameter that accounts for several simultaneous moisture transfer mechanisms, which influence the drying process in different ways. Thus, it is an oversimplification to represent this complex set of mechanisms in only a few parameters.^36^

**Figure 2:**
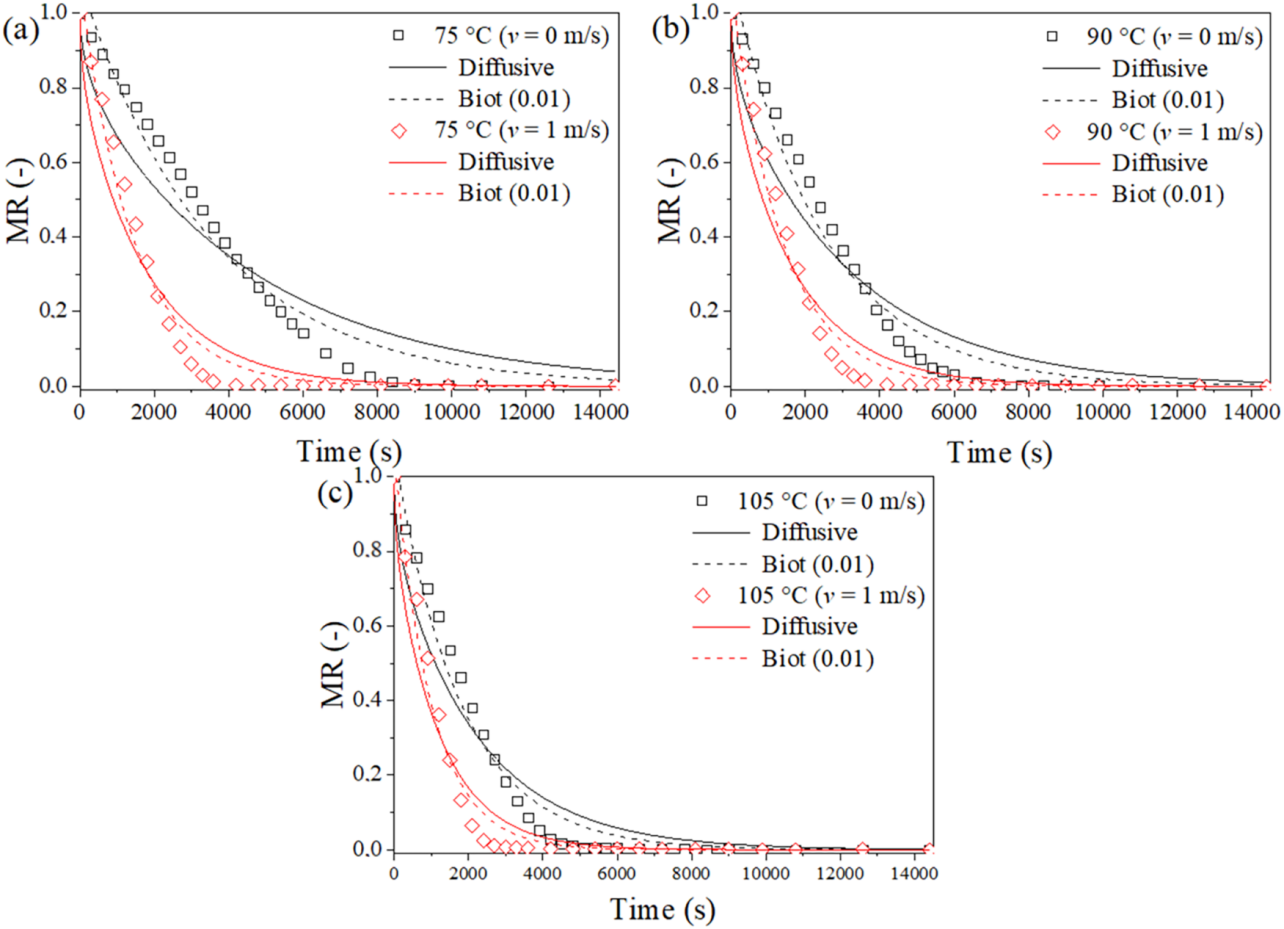
Experimental drying kinetics data and predictions obtained from the one- and two-parameter diffusive models, with Biot number (*Bi*_m_ = 0.01), for dry LDHs at different temperatures: (a) 75 °C, (b) 90 °C, and (c) 105 °C.

There are no results in the literature about effective diffusivity for LDH drying. For agricultural and food materials, *D*_eff_ values lie within a magnitude range of 10^–11^ to 10^–9^ m^2^/s.^37–40^ For inorganic materials, such as alumina, *D*_eff_ values ranging from 10^–8^ to 10^–6^ m^2^/s were found.^36,41^ The values obtained in this study, particularly under conditions where *Bi*_m_ approaches zero, fall within the typical range reported for inorganic materials.

### Activation Energy

Table 3 presents the activation energy values and the corresponding coefficients of determination. The highest activation energy values were observed under conditions where internal resistance governs the drying process, reaching 26.805 kJ/mol for the one-parameter diffusive model and 26.794 kJ/mol when *Bi*_m_ tends toward infinity. Conversely, the lowest energy activation values, ranging from 12.547 to 12.577 kJ/mol, were obtained under conditions characterized by low *Bi*_m_, where external mass transfer resistance governs the process.

**Table 3:** Activation Energy for Oven Drying (*E*_a_ = kJ/mol)

	without convection		with convection	
model	*E*_a_	*R* ^2^	*E*_a_	*R* ^2^
diffusive	26.80	0.9991	13.92	0.8273
*Bi*_m_ 0.01	24.25	0.9999	12.58	0.8211
*Bi*_m_ 0.1	24.25	0.9999	12.57	0.8213
*Bi*_m_ 1	24.22	0.9999	12.55	0.8210
*Bi*_m_ 100	26.62	0.9991	13.87	0.8280
*Bi*_m_ Inf	26.79	0.9991	13.93	0.8273

As for effective diffusivity, there are no activation energy results for LDH drying. Activation energy magnitudes for agricultural and food products are generally reported to be between 13 and 110 kJ/mol,^25,38,42^ where more than 90% are in the range of 14.42 and 43.26 kJ/mol.^38,43^ Due to their structures, external coatings, internal characteristics, and compositions, food and agricultural products have relatively high energy activation values, with a drying rate governed by internal mass transfer (*Bi*_m_ > 50).

The low activation energies observed, particularly in the presence of convection, indicate that the surface diffusion of liquid molecules along the capillary walls is the limiting mass transfer mechanism in the drying process. This implies that moisture is primarily transported along capillary walls through chemical and physical interactions with the solid. In the constant drying rate, the predominant phase in the drying of LDHs (Figure 1c), the heat supplied by the drying air is used to evaporate surface water. At the same time, internal diffusion contributes only secondarily to replenishing it. When the liquid film breaks and most of the surface is exposed, the falling-rate phase begins, controlled by combined transport (capillary flow, liquid diffusion in the pores, and vapor flow). As drying progresses, capillary tensions and adsorption forces between water and the LDH structure increase, making moisture removal more difficult and reducing the drying rate. In this regime, the evaporation rate becomes less sensitive to external conditions and is governed by internal diffusive mechanisms of liquid and vapor transport (Figure 3).^19,21,29,33^

**Figure 3:**
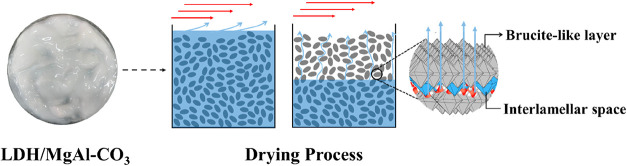
Diagram of the drying of LDH/MgAl-CO_3_, illustrating water removal by capillary transport and diffusion.

Due to the pressure difference caused by capillary pressure, along with excess evaporation that can occur at the beginning of the drying process, several problems can occur with the material, such as the disappearance of micropores and structural deformation, resulting in large agglomerations (Figure S.3). Since LDH is an inorganic material that does not undergo significant structural changes at temperatures below 180 °C,^4,44,45^ the surface diffusion of the molecules, established from a concentration gradient, is expected. Thus, free water is basically removed in the drying step at a constant rate, and there is no significant barrier to transport by ordinary diffusion through the particle.

## Conclusions

A comprehensive understanding of the drying behavior of layered double hydroxides (LDHs) is crucial for the controlled and efficient production of these materials. In this study, the drying rate of MgAl–CO_3_/LDH was evaluated, and the distinct drying stages were characterized. The effective moisture diffusivity ranged from 9.53 × 10^–10^ to 1.16 × 10^–7^ m^2^/s, while the energy activation varied between 12.55 and 26.80 kJ/mol, depending on the drying conditions and the dominant mass transfer mechanisms.

These findings provide valuable insights into the mass transport phenomena during LDH drying and establish a quantitative basis for optimizing drying parameters. Moreover, the results presented herein offer a foundational reference for future investigations involving alternative drying techniques, such as spray drying, freeze-drying, and microwave-assisted drying.

## Supplementary Material



## Notes

Additional experimental details for SEM and XRD; XRD patterns of LDHs dried at 75, 90, and 105 °C; representative SEM micrographs of LDHs obtained at these temperatures; drying evolution profile of LDH at 90 °C (PDF)

## ∥

Bioenergy and Energy Efficiency Laboratory, Institute of Technological Research of the State of São Paulo (IPT), São Paulo, São Paulo 05508901, Brazil
