# Supplementary material for: Mathematical Modeling of LDH Nanoparticle Drying: Evaluating Effective Diffusivity and the Role of the Mass Biot Number
Source: ACS Omega. 2025 Dec 20;11(1):1867–73. doi: 10.1021/acsomega.5c10016 (PMC12809506; doi:10.1021/acsomega.5c10016)
Supplement: Supplementary file 1 [file ao5c10016_si_001.pdf]

Mathematical Modeling of LDH Nanoparticle Drying: Evaluating Effective Diffusivity and the Role of the Mass Biot Number

Luiz D. Silva Neto[1], Rodolfo Junqueira Brandão[2], Thais Logetto Caetité Gomes[2,3], Lucas Meili[3], José Teixeira Freire[1]

[1]Drying Center of Pastes, Suspensions, and Seeds, Department of Chemical Engineering, Federal University of São Carlos (UFSCar), São Carlos, São Paulo, 13565-905, Brazil

[2] Flowlab (Fluid Dynamics Laboratory), Center of Technology, Federal University of Alagoas, Av. Lourival Melo Mota, s/n, Campus A.C. Simões, Tabuleiro do Martins, Maceió-AL, 57072-970, Brazil.

[3]Laboratory of Processes, Center of Technology, Federal University of Alagoas, Av. Lourival Melo Mota, s/n, Campus A.C. Simões, Tabuleiro do Martins, Maceió-AL, 57072-970, Brazil.

Corresponding author: Lucas Meili, Dr., lucas.meili@ctec.ufal.br. Laboratory of Processes, Center of Technology, Federal University of Alagoas, Av. Lourival Melo Mota, s/n, Campus A.C. Simões, Tabuleiro do Martins, Maceió-AL, 57072-970, Brazil.

S.1. Material characterization

The microstructural analyses of the materials were performed by scanning electron microscopy (SEM) images on a FEI microscope (model Inspect S 50) with an accelerating voltage of 20 kV and a current of 10 mA. The samples were sprinkled onto a carbon-conductive double-sided tape and covered with conductive material using the gold film deposition system (Desk V, Denton Vacuum LLC). X-ray diffraction (XRD) analysis of all materials was performed on a Rigaku Multiflex diffractometer using the powder method with CuKα radiation (λ = 1.5406 Å; 40 kV-15 mA), scanning 2θ° min^{-1} (2° to 90°) and a step size of 0.02 θ°.

S.2. X-ray diffraction (XRD)

The XRD patterns of the LDHs synthesized with the three different dryers are presented in Figure S.1. The materials presented symmetric reflections for the planes 2θ = 11.56° (003), 23.20° (006), 34.70° (009/012), 60.76° (110) and 62.22° (113), and asymmetric reflections for the non-basal planes 2θ = 34.70° (009/012), 38.16° (015) and 46.52° (018), indicating that a hydrotalcite-like structure was formed [1].

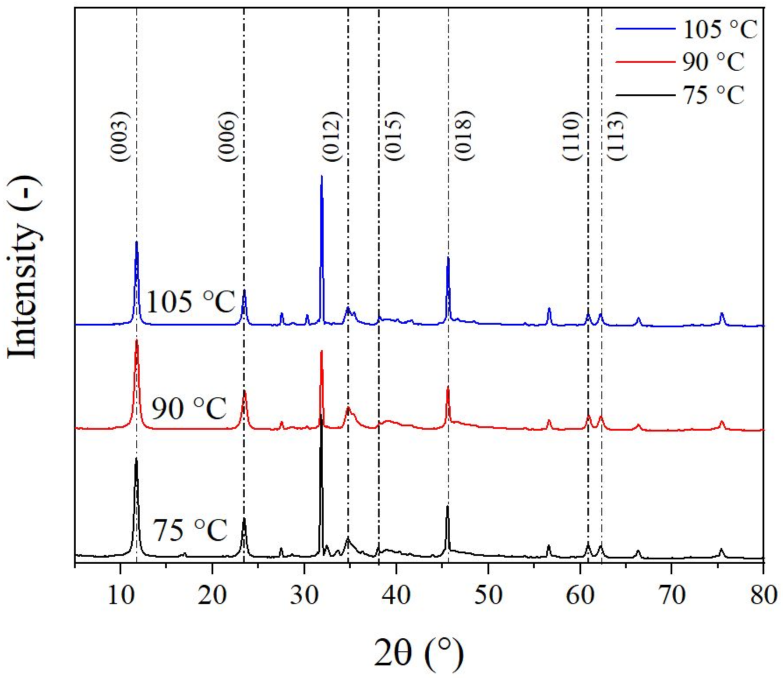

Figure S.1. X-ray diffractogram of dried LDHs at different temperatures: (a) 75 °C, (b) 90 °C, and (c) 105 °C.

S.3. Scanning Electron Microscopy (SEM)

Figure S.2 shows the morphology of the materials obtained by scanning electron microscopy for the experiments performed in the different dryers. The images present distinct, better-defined, and angled shapes of varying sizes.

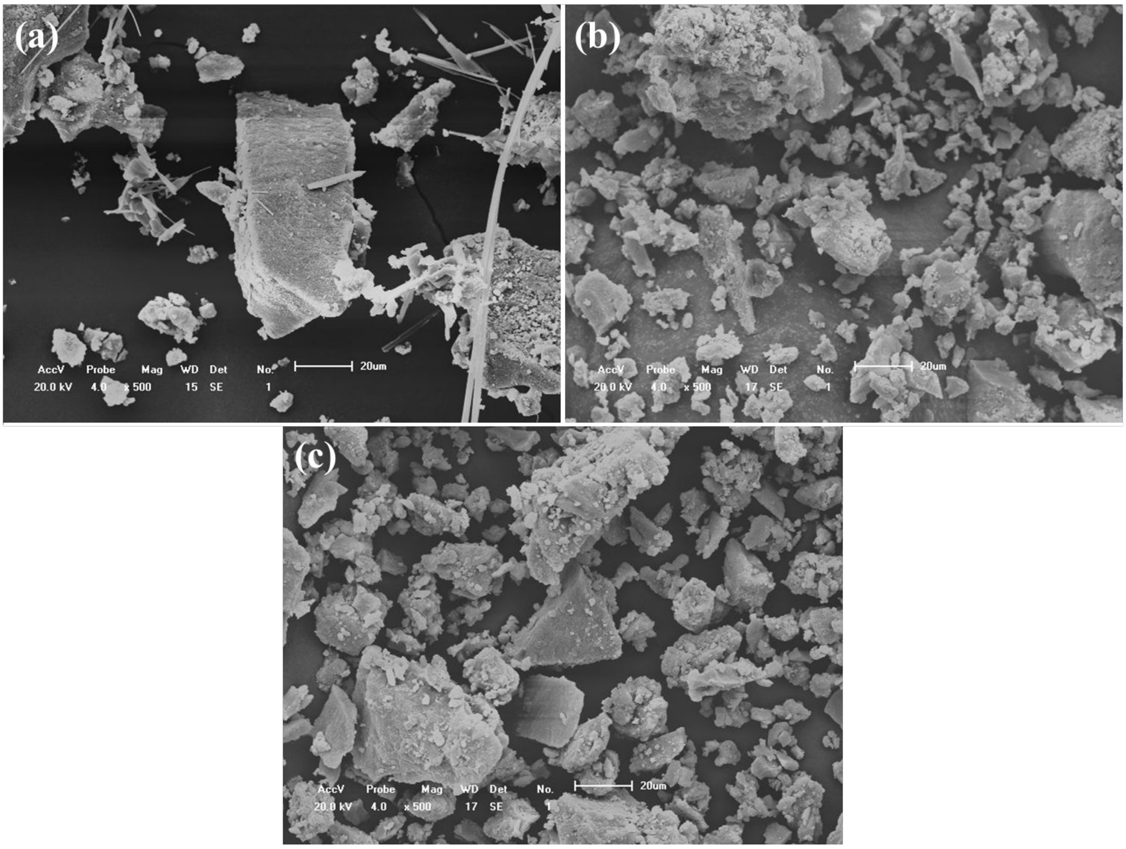

Figure S.2. Representative scanning electron microscopy images of dried LDHs at different temperatures: (a) 75 °C, (b) 90 °C, and (c) 105 °C.

S.4. Evolution of LDH drying

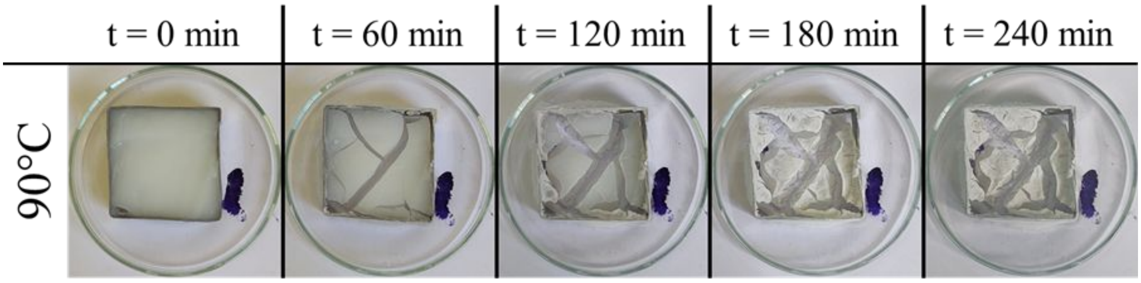

Figure S.3. Evolution of LDH drying at a temperature of 90 °C.

References

[1] A. V. Radha, P.V. Kamath, C. Shivakumara, Conservation of order, disorder, and "crystallinity" during anion-exchange reactions among Layered Double Hydroxides (LDHs) of Zn with Al, Journal of Physical Chemistry B 111 (2007) 3411–3418. https://doi.org/10.1021/jp0684170.
